# Expression analyses of the genes harbored by the type 2 diabetes and pediatric BMI associated locus on 10q23

**DOI:** 10.1186/1471-2350-13-89

**Published:** 2012-09-24

**Authors:** Jianhua Zhao, Sandra Deliard, Ali Rahim Aziz, Struan FA Grant

**Affiliations:** 1 Division of Human Genetics, The Children’s Hospital of Philadelphia, Philadelphia, USA; 2 Department of Pediatrics, University of Pennsylvania School of Medicine, Philadelphia, USA

**Keywords:** Obesity, Pediatrics, Expression, Genomics

## Abstract

**Background:**

There is evidence that one of the key type 2 diabetes (T2D) loci identified by GWAS exerts its influence early on in life through its impact on pediatric BMI. This locus on 10q23 harbors three genes, encoding hematopoietically expressed homeobox (*HHEX*)*,* insulin-degrading enzyme (*IDE*) and kinesin family member 11 (*KIF11*), respectively.

**Methods:**

We analyzed the impact of adipogeneis on the mRNA and protein expression levels of these genes in the human adipocyte Simpson-Golabi-Behmel syndrome (SGBS) cell line in order to investigate which could be the culprit gene(s) in this region of linkage disequilibrium.

**Results:**

Following activation of differentiation with a PPARγ ligand, we observed ~20% decrease in *IDE*, ~40% decrease in *HHEX* and in excess of 80% decrease in *KIF11* mRNA levels when comparing the adipocyte and pre-adipocyte states. We also observed decreases in KIF11 and IDE protein levels, but conversely we observed a dramatic increase in HHEX protein levels. Subsequent time course experiments revealed some marked changes in expression as early as three hours after activation of differentiation.

**Conclusion:**

Our data suggest that the expression of all three genes at this locus are impacted during SGBS adipogenesis and provides insights in to the possible mechanisms of how the genes at this 10q23 locus could influence both adipocyte differentiation and susceptibility to T2D through insulin resistance.

## Background

Genome wide association studies (GWAS) have revealed robust signals for both body mass index (BMI) and type 2 diabetes (T2D) [1-5]. In the case of the strongest associated loci with BMI and T2D to date, namely *FTO*[6] and *TCF7L2*[7] respectively, they are the only genes harbored in their respective regions of linkage disequilibrium where the GWAS signal resides, making them obvious candidates for the culprit gene at these given locations. However this is not always the case for the other GWAS loci reported, most notably the multi-gene 10q23 T2D locus [1].

We previously investigated in a pediatric setting all the main loci identified by T2D GWAS and reported that only the 10q23 variant allele was also associated with increased BMI in childhood, suggesting that it exerts its influence early on in life [8]. In addition, this locus has also been reported to be associated with BMI in children at eight years of age who were born large for gestational age to a parent with type 1 diabetes [9].

The 10q23 locus contains three genes within the region of linkage disequilibrium harboring the T2D and BMI associated signal, encoding insulin-degrading enzyme (*IDE*), kinesin family member 11 (*KIF11*) and hematopoietically expressed homeobox (*HHEX*)*,* respectively down the chromosomal arm, with *KIF11* and *HHEX* transcribed on the sense strand. Based on the observations that this locus impacts BMI early in life, we hypothesized that the culprit gene(s) at this locus may in fact impact the adipogenesis process. We elected to use the Simpson-Golabi-Behmel syndrome (SGBS) cell line, commonly used to study human adipocyte biology [10-13], to investigate the expression of these three genes at the mRNA and protein level during cell adipogenesis. This in turn allowed us to test our hypothesis and to investigate if we could determine which gene(s) in this region was the culprit with respect to the previously reported association with T2D and pediatric BMI.

## Methods

### Cell culture

SGBS cells were obtained from Dr. Martin Wabitsch’s lab at the University of Ulm [12]. Culturing the pre-adipocyte state of the SGBS cells and the subsequent differentiation of the cells to adipocytes was carried out as previously described [12]. Cells were considered pre-adipocytes before the addition of the differentiation medium and were subsequently considered adipocytes once lipidation was observed. The percentage of differentiation was 90%, as determined by counting the number of cells containing lipid droplet versus the cells without lipid droplet. Either Troglitazone (Sigma Inc) or Rosiglitazone (Cayman Chemicals) was used as the PPARγ ligand in the culture medium (0.008 mg/ml biotin, 0.004 mg/ml panthanoate, 1% penicillin/streptomycin, 10 mg/L transferrin, 1uM insulin, 0.2nM T3, 250nM Dexamethasone, 500uM IBMX and 2uM Rosiglitazone or Troglitazone). Cell images were captured using a Zeiss confocal microscope.

### Quantitative polymerase chain reaction

RNA was purified with the RNAeasy kit (Qiagen Inc) and then the cDNA was synthesized using the Advantage RT-for-PCR kit (Clontech laboratories). Fully quantitative PCR was carried out using Power SYBR green qPCR mastermix (Applied Biosystems Inc) on the 7900HT real-time PCR system (Applied Biosystems Inc). All primers ( Additional file 1: Table S1) had a single peak in the dissociation curve following PCR. The Day 0 value was arbitrarily set at 1. At least three independent experiments were performed, of which the mean +/−S.D. is presented.

### Western blot

Whole cell protein was extracted in RIPA (Radio-immunoprecipitation Assay) buffer (150 mM NaCl, 1% IGEPAL CA-630, 0.5% sodium deoxycholate, 0.1%SDS, 50 mM Tris–HCl, pH8.0). Protein concentrations were measured by BCA protein assay kit (Thermo Scientific) as described previously [14]. Proteins were separated in a Nupage 10% BT gel (Invitrogen Inc) and transferred to a polyvinylidene difluoride (PVDF) membrane (Amersham). The chemiluminescent reagent, ECL plus (Amersham), was used for detection. The primary antibodies used in this study were for HHEX (ab79392, Abcam), KIF11 (Abcam), IDE (Thermo Scientific), RAN (BD biosciences) and 36B4 (Abcam). At least three independent experiments were performed, with representative figures presented.

### Loading controls

Loading controls used commonly, such as GAPDH and actin, change greatly during the adipogenesis process perhaps reflecting structural changes in the cell during adipogenesis. RAN was used as a loading control by Schupp M *et al.*[15]. Similar to RAN, 36B4 is a nuclear protein. In our experience, both RAN and 36B4 decrease slightly during adipogenesis, the level of change is trivial comparing to the change we observed with HHEX and KIF11. Thus we used RAN and 36B4 interchangeably as loading controls in these experiments to illustrate the change in HHEX and KIF11 protein expression levels.

### PPARG agonists

Both rosiglitazone and troglitazone are PPARG agonists so the fact that we observed similar results using both ligands further supports our results. Rosiglitazone is typically a more potent ligand than troglitazone, and it takes a shorter time for lipids to form. Rosiglitazone and troglitazone are often used interchangeably [16].

## Results

### Comparison of mRNA and protein expression levels between the pre-adipocyte and adipocyte stages of SGBS cells

**Figure 1:**
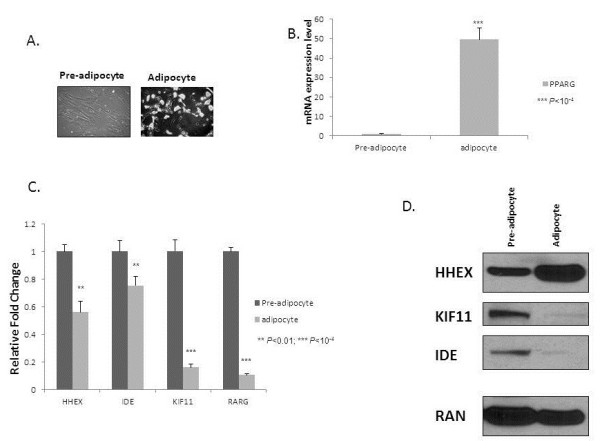
**Expression of the three genes within the 10q23 locus at both the pre-adipocyte and adipocyte stage of SGBS cell differentiation. **mRNA and protein were extracted from SGBS cells either at the pre-adipocyte or adipocyte stage cultured in differentiation medium supplemented with Troglitazone for 27–49 days. Confocal image of SGBS cells in the pre-adipocyte and adipocyte states (49 days in differentiation medium) are shown (**A**). In (**B**) change in real-time PCR normalized for *36B4* mRNA expression levels of the *PPARG* control before and after differentiation; as expected, *PPARG* levels increase. In (**C**), change in real-time PCR normalized for *36B4* mRNA expression levels in *HHEX*, *IDE,* and *KIF11* mRNA expression levels; the levels of the *RARG* control decrease as expected. Western blots (**D**) were carried out to measure either mRNA and protein levels of the genes of interest, respectively. Standard deviations were calculated based on four independent mRNA replicates. A representative Western blot result is presented.

The SGBS cells were cultured in differentiation medium supplemented with troglitazone for 27–49 days. After 49 days, accumulation of lipid droplets was observed indicating a mature adipocyte state (Figure 1A). The mRNA and protein isolations were carried out in parallel but in separate culture dishes.

For the mRNA expression analyses, *PPARγ* and *RARγ* were used as positive controls [17]. The housekeeping gene, *36B4*, was used as a normalization control. There was indeed a marked increase (>50 fold) of *PPARγ* mRNA in adipocytes compared to the pre-adipocyte state (Figure 1B) and an equally marked decrease (~90%) of *RARγ* mRNA levels (Figure 1C). Combining the confocal and mRNA evidence from the *PPARγ* and *RARγ* data, it was clear that these cells underwent differentiation to adipocyte as expected. We observed an approximate 20% decrease in *IDE*, 40% decrease in *HHEX* and a greater than80% decrease in *KIF11* mRNA levels when comparing the adipocyte state with the pre-adipocyte state.

For the parallel protein level analyses, we used ubiquitously expressed GTPase RAN as the loading control [15]. Similar to the mRNA data, we observed decreases in KIF11 and IDE protein levels; on the other hand, and converse to our mRNA observations, we observed a dramatic increase in HHEX protein levels (Figure 1D).

### Time course expression analyses during SGBS differentiation

We went on to examine the expression of *HHEX*, *IDE* and *KIF11* during the course of SGBS pre-adipocyte differentiation to adipocytes. We carried out the time courses in differentiation medium supplemented with a PPARγ agonist, either being 2uM troglitazone or rosiglitazone. The results were highly comparable, with any slight differences primarily due to potency differences of the PPARγ agonist ( Additional file 2: Figure S1).

**Figure 2:**
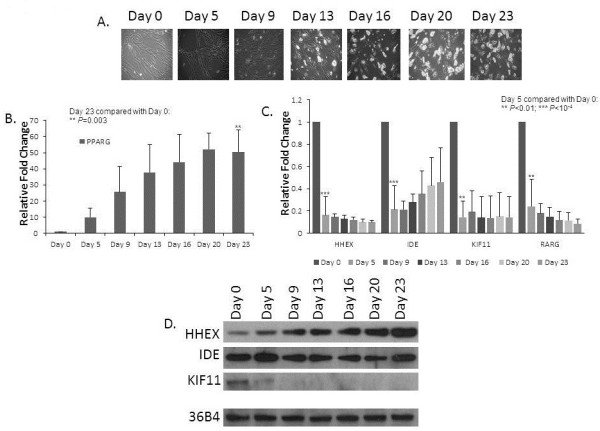
**Expression time course of the three genes within the 10q23 locus during adipogenesis in SGBS cells.** mRNA and protein were extracted from SGBS cells at given time points (Days 0, 5, 9, 13, 16, 20 and 23) following introduction of the differentiation medium supplemented with Rosiglitazone. Confocal images of the SGBS cells are presented at each of these time points (**A**). Real-time PCR normalized for *36B4* mRNA expression (**B**, **C**) and Western blots (**D**) were carried out to measure either mRNA or protein levels of the gene of interest, respectively. Standard deviations were calculated based on three independent mRNA replicates. A representative Western blot result is presented.

Three independent differentiation assays were carried out using rosiglitazone as the PPARγ agonist (Figure 2). After adding the differentiation medium, we observed increased lipid droplet accumulation over time as expected (Figure 2A); in addition, we observed the expected gradual increase of *PPARγ* mRNA (Figure 2B) and protein (data not shown) expression. As seen in Figure 2C, as early as 5 days after adding the differentiation medium, along with the control *RARγ* we observed a marked drop in the mRNA levels of *HHEX*, *IDE* and *KIF11*; however, the expression patterns for the four genes differed over time, with *HHEX*, *KIF11* and *RARγ* mRNA levels remaining low after day 5, while the *IDE* mRNA levels rebounded to some degree from day 13 to day 23.

We also examined protein levels in the presence of either troglitazone or rosiglitazone in the differentiation medium, with PPARγ serving as a positive control and 36B4 serving as loading controls (Figure 2D and Additional file 2: Figure S2). Again, in contrast to the *HHEX* mRNA data, we observed a gradual increase of HHEX protein levels, starting as early as 5 days after adding the differentiation medium and continuing to increase until day 23. We observed a decrease of KIF11 protein levels from day 5 while protein levels for IDE largely remained steady over time following a relatively modest drop after Day 5.

### Expression during early adipogenesis of SGBS cells

As much of the expression differences occurred early on in the time course experiments, we elected to explore the mRNA and protein levels within 5 days of adding differentiation medium. We observed an immediate drop of *HHEX* mRNA expression, while we observed a relatively more gradual drop of *IDE*, *KIF11* and *RARγ* levels and a gradual increase in *PPARγ* levels. In order to ascertain that the dramatic *HHEX* mRNA data were not biased due to uncharacterized spliced isoforms, we designed a second pair of primers targeting the 5’ end of the gene (as opposed to the 3’ end for the first primer pair) and found the data to be consistent ( Additional file 2: Figure S3A and Additional file 2: Figure S4).

At the protein level, we explored HHEX and KIF11 expression in this short time period, as there was previous evidence they changed noticeably between Day 0 and Day 5 (Figure 2 and Additional file 2: Figure S2).We observed an immediate increase in HHEX protein levels while there was a relatively gradual decrease of KIF11 protein levels within Day 1 and then dropped off in Day 2 onwards ( Additional file 2: Figure S3B).

## Discussion

Our results point to all three genes within the 10q23 BMI and T2D associated locus having their expression strongly influenced during adipogenesis. The most notable outcome from our experiments is with HHEX, where mRNA levels drop dramatically early on in the adipogenesis process while, conversely, there is a steady increase in protein levels during the same period. In addition, both mRNA and protein levels for the *KIF11* gene product drop quickly after the start of adipogenesis while the protein levels for the *IDE* gene product remain relatively stable during the same period, although the mRNA levels drop quickly after the start of the process followed by a rebound.

The known functions of these three genes have little in common. *KIF11* encodes a motor protein [18], *IDE* encodes a protease [19] and *HHEX* encodes a transcription factor. As such, their possible coordinated modes of action would require further investigation.

The fact that we do not observe a direct correlation with *HHEX* mRNA expression and protein levels may point to protein stability differences between the pre-adipocyte and adipocytes states, or conversely the mRNA may degrade over time or perhaps, more specifically, just a subset of the splice isoforms. Alternatively, the level by cell volume / surface area could be less in adipocytes compared to pre-adipocytes since adipocytes expand in cell volume during adipogenesis. As such, further studies are warranted through protein stability and cell-based assays to fully understand the mechanisms of action in this regard.

## Conclusions

Our data provide insights in to the possible mechanisms of how all the genes at the T2D and pediatric BMI associated 10q23 locus could influence adipocyte differentiation. Our conclusion is that all three genes are important in this region for the expression of this trait and we were unable to determine the ‘culprit’ gene as there may well be not only one in this region. Further studies are required to examine the possible mechanisms of action suggested by our analyses.

## Competing interests

The authors declare that they have no competing interests.

## Authors’ contributions

JZ and SFAG designed the study and supervised the data analysis and interpretation. JZ, SD and ARA conducted the cell work and expression analyses. JZ and SFAG conducted the statistical analyses. JZ, SD and SFAG drafted the manuscript. All authors read and approved the final manuscript.

## Disclosure statement

All authors have nothing to disclose.

## Grant support

The study is supported by an Institute Development Award from The Children’s Hospital of Philadelphia and NIH grant R01 HD056465.

## Pre-publication history

The pre-publication history for this paper can be accessed here:

http://www.biomedcentral.com/1471-2350/13/89/prepub

## Supplementary Material

Additional file 1 **Table S1. **Primers used in the quantitative PCR experiments. Click here for file

Additional file 2 **Figure S1. **Compararison of the expression of the genes within the 10q23 locus using either Troglitazone or Rosiglitazone as the PPARγ agonist during adipogenesis in SGBS cells; **Figure S2.** Western blot data for the three genes within the 10q23 locus during SGBS cell adipogenesis using Troglitazone as the PPARγ agonist; Figure S3. Expression time course of the three genes within the 10q23 locus during early adipogenesis in SGBS cell; Figure S4. Consistency of *HHEX* real-time PCR result utilizing two primer sets. Click here for file
