# Supplementary material for: Expression analyses of the genes harbored by the type 2 diabetes and pediatric BMI associated locus on 10q23
Source: BMC Med Genet. 2012 Sep 24;13:89. doi: 10.1186/1471-2350-13-89 (PMC3514277; doi:10.1186/1471-2350-13-89)
Supplement: Additional file 1 — Table S1. Primers used in the quantitative PCR experiments. [file 1471-2350-13-89-S1.doc]

**Supplementary Table 1: Primers used in the quantitative PCR experiments**

HHEX_F: GCGAGAGACAGGTCAAAACC

HHEX_R: AGGGCGAACATTGAGAGCTA

HHEX2_F: CGGACGGTGAACGACTACAC

HHEX2_R: TTCTTCTCCAGCTCGATGGT

IDE_F: ATGTTCTTGCCAGGGAAATG

IDE_R: CAGAGTTTTGCAGCCATGAA

KIF11_F: GGCAGTTGACCAACACAATG

KIF11_R: TCTAGCATGGCCTTTTGCTT

PPARγ_F: CGACCAAGTAACTCTCCTCA

PPARγ_R: GTTCCGTGACAATCTGTCTG

RARγ_F: CTCACAGACCTTGTCTTTGC

RARγ_R: CTGGAATCTCCATCTTCAGAG

36B4_F: GATGCCCAGGGAAGACAG

36B4_R: TTGAGGACCTCTGTGTATTTGTCAA
