# Supplementary material for: Expression analyses of the genes harbored by the type 2 diabetes and pediatric BMI associated locus on 10q23
Source: BMC Med Genet. 2012 Sep 24;13:89. doi: 10.1186/1471-2350-13-89 (PMC3514277; doi:10.1186/1471-2350-13-89)
Supplement: Additional file 2 — Figure S1. Compararison of the expression of the genes within the 10q23 locus using either Troglitazone or Rosiglitazone as the PPARγ agonist during adipogenesis in SGBS cells; Figure S2. Western blot data for the three genes within the 10q23 locus during SGBS cell adipogenesis using Troglitazone as the PPARγ agonist; Figure S3. Expression time course of the three genes within the 10q23 locus during early adipogenesis in SGBS cell; Figure S4. Consistency of HHEX real-time PCR result utilizing two primer sets. [file 1471-2350-13-89-S2.ppt]

## Slide 1
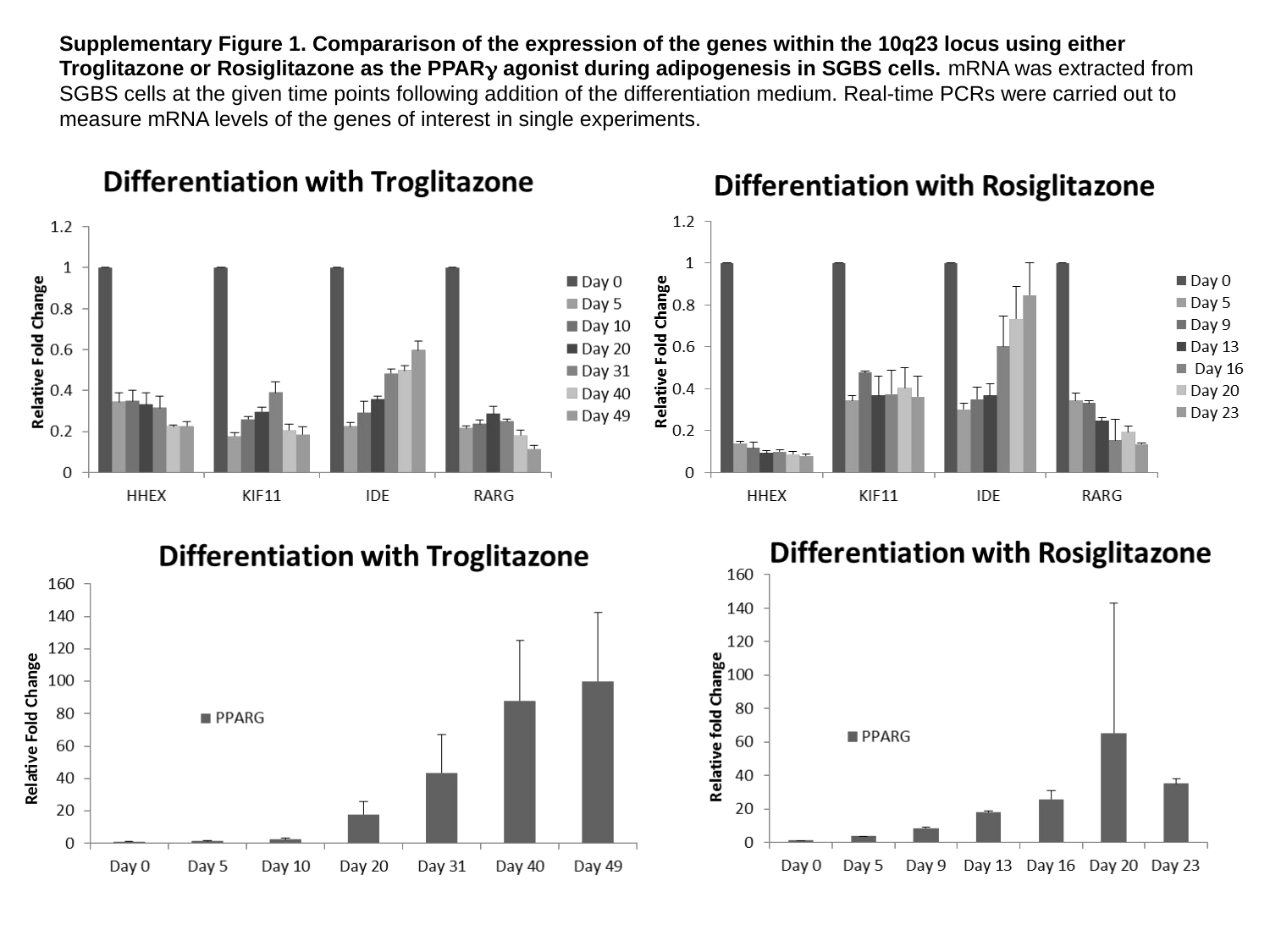

Supplementary Figure 1. Compararison of the expression of the genes within the 10q23 locus using either Troglitazone or Rosiglitazone as the PPARγ agonist during adipogenesis in SGBS cells. mRNA was extracted from SGBS cells at the given time points following addition of the differentiation medium. Real-time PCRs were carried out to measure mRNA levels of the genes of interest in single experiments.
Relative Fold Change

## Slide 2
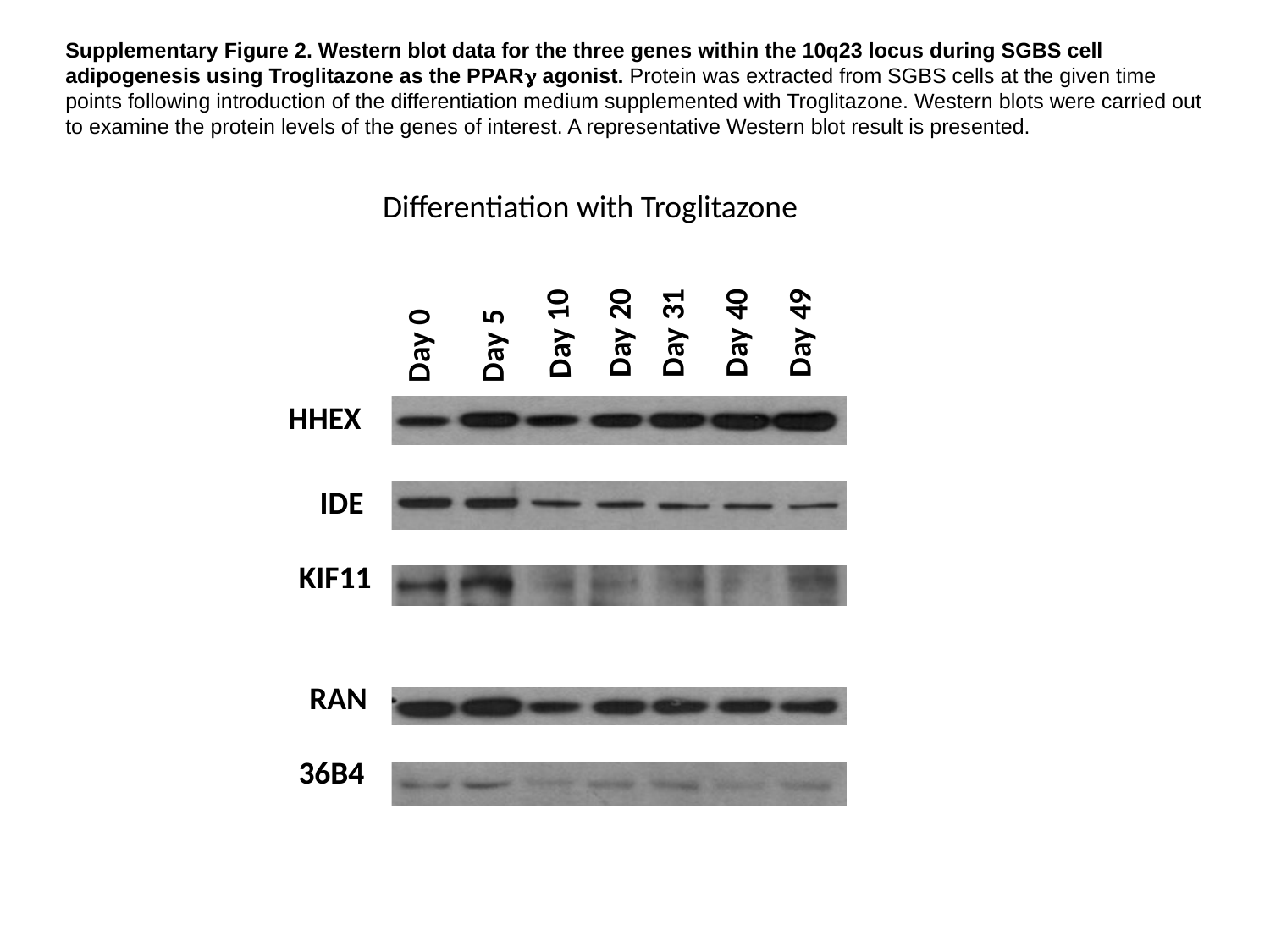

Supplementary Figure 2. Western blot data for the three genes within the 10q23 locus during SGBS cell adipogenesis using Troglitazone as the PPARγ agonist. Protein was extracted from SGBS cells at the given time points following introduction of the differentiation medium supplemented with Troglitazone. Western blots were carried out to examine the protein levels of the genes of interest. A representative Western blot result is presented.
Differentiation with Troglitazone
Day 20
Day 31
Day 40
Day 49
Day 10
Day 0
Day 5
HHEX
IDE
KIF11
RAN
36B4

## Slide 3
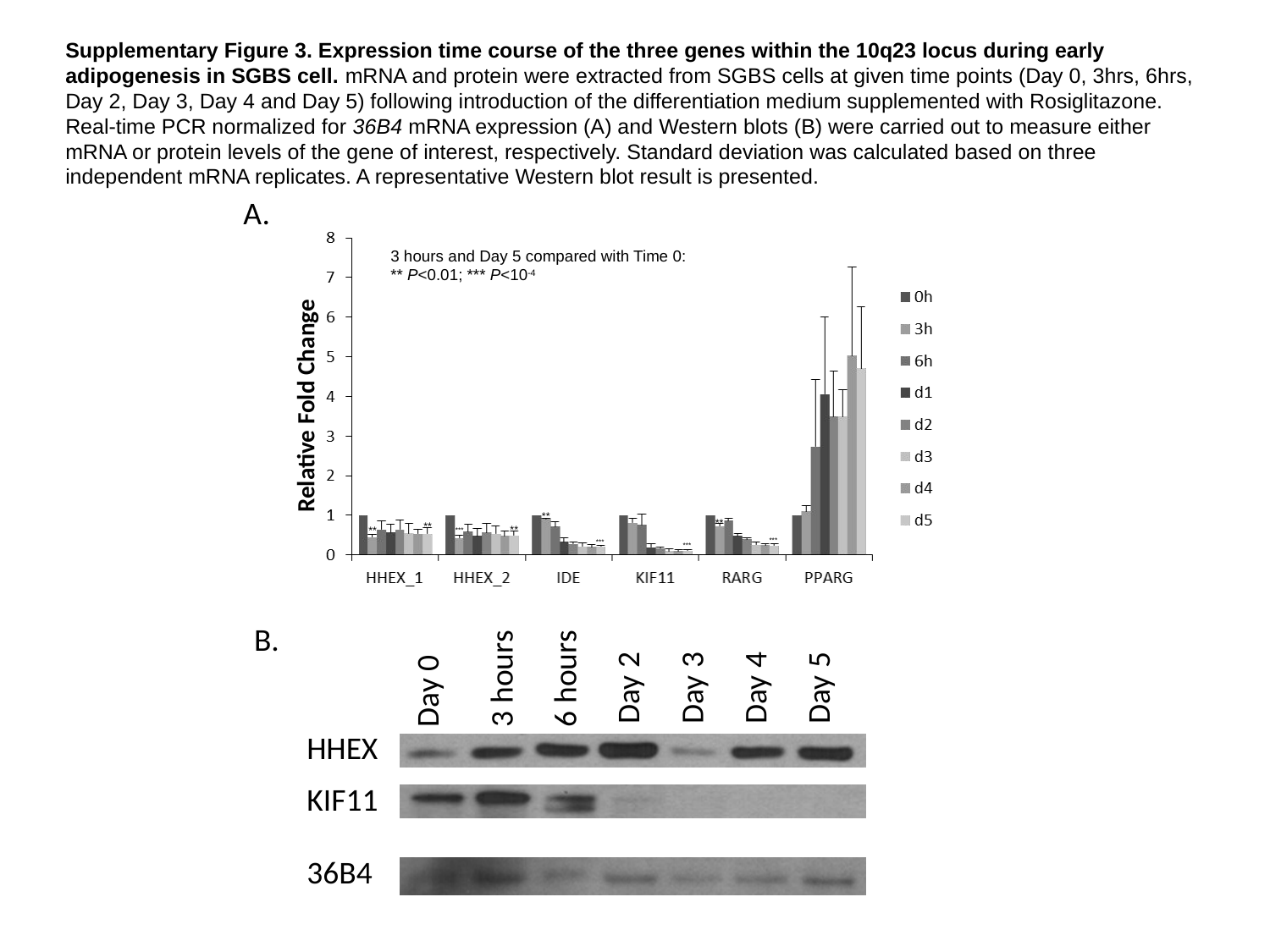

Supplementary Figure 3. Expression time course of the three genes within the 10q23 locus during early adipogenesis in SGBS cell. mRNA and protein were extracted from SGBS cells at given time points (Day 0, 3hrs, 6hrs, Day 2, Day 3, Day 4 and Day 5) following introduction of the differentiation medium supplemented with Rosiglitazone. Real-time PCR normalized for 36B4 mRNA expression (A) and Western blots (B) were carried out to measure either mRNA or protein levels of the gene of interest, respectively. Standard deviation was calculated based on three independent mRNA replicates. A representative Western blot result is presented.
A.
3 hours and Day 5 compared with Time 0:
** P<0.01; *** P<10-4
Relative Fold Change
**
**
**
**
**
***
***
***
***
Day 4
B.
Day 3
Day 5
3 hours
Day 2
6 hours
Day 0
HHEX
KIF11
36B4

## Slide 4
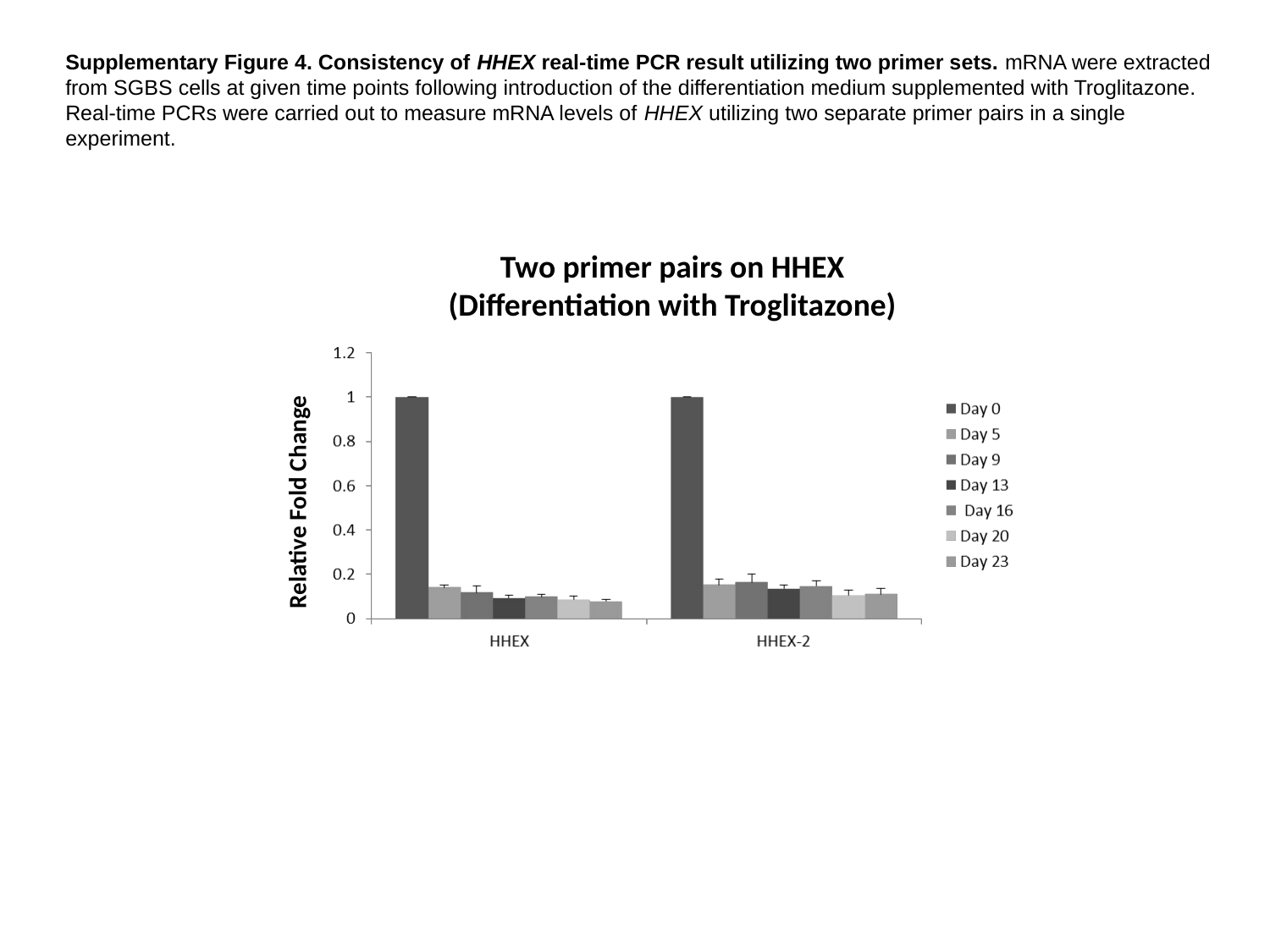

Supplementary Figure 4. Consistency of HHEX real-time PCR result utilizing two primer sets. mRNA were extracted from SGBS cells at given time points following introduction of the differentiation medium supplemented with Troglitazone. Real-time PCRs were carried out to measure mRNA levels of HHEX utilizing two separate primer pairs in a single experiment.
Two primer pairs on HHEX (Differentiation with Troglitazone)
Relative Fold Change
